# A New Steel

**Published:** 1884-04

**Authors:** 


A NEW STEEL is said to have been produced at Sheffield, England, which is expected to be of incalculable value to the manufacturing and railroad world. It is said to be made "by adding from seven to twenty per cent. of the ordinary ferro-maganese of commerce to iron either wholly or to a good extent decarbonized and refined and treated by any of the ordinary processes, or to steel produced by such processes." It is stated that a small test bar containing twelve per cent. of maganese was bent double when cold, and was sufficiently hard to turn iron; that an axe containing the same percentage, and which had never been hardened or tempered, cut in two a bar of iron half an inch square. A correspondent of *The American Manufacturer*, giving these facts, says that the steel is capable of being hammered or rolled the same as ordinary steel, and showed no magnetic qualities. If these accounts are in any measure correct, the discovery is likely to prove of great economic importance.

